# Dissecting the Complexity of Early Heart Progenitor Cells

**DOI:** 10.3390/jcdd9010005

**Published:** 2021-12-26

**Authors:** Miquel Sendra, Jorge N. Domínguez, Miguel Torres, Oscar H. Ocaña

**Affiliations:** 1 Developmental Biology Program, Centro Nacional de Investigaciones Cardiovasculares (CNIC), 28029 Madrid, Spain; mtorres@cnic.es; 2 Cardiovascular Development Group, Department of Experimental Biology, University of Jaen, 23071 Jaen, Spain; jorgendm@ujaen.es

**Keywords:** cardiac fields, cardiomyocyte, endocardium, progenitor specification, heart tube

## Abstract

Early heart development depends on the coordinated participation of heterogeneous cell sources. As pioneer work from Adriana C. Gittenberger-de Groot demonstrated, characterizing these distinct cell sources helps us to understand congenital heart defects. Despite decades of research on the segregation of lineages that form the primitive heart tube, we are far from understanding its full complexity. Currently, single-cell approaches are providing an unprecedented level of detail on cellular heterogeneity, offering new opportunities to decipher its functional role. In this review, we will focus on three key aspects of early heart morphogenesis: First, the segregation of myocardial and endocardial lineages, which yields an early lineage diversification in cardiac development; second, the signaling cues driving differentiation in these progenitor cells; and third, the transcriptional heterogeneity of cardiomyocyte progenitors of the primitive heart tube. Finally, we discuss how single-cell transcriptomics and epigenomics, together with live imaging and functional analyses, will likely transform the way we delve into the complexity of cardiac development and its links with congenital defects.

## 1. Introduction

The heart is the first organ to form during embryogenesis. Following the onset of gastrulation, a primitive heart assembles and starts pumping nutrients to the whole embryo, while it continues developing. This remarkable ability to form and function simultaneously has attracted researchers for nearly a century, revealing that heart development involves the interplay between heterogeneous cell sources and complex morphogenetic processes [1]. With at least 0.8% of newborns presenting congenital heart defects [2], understanding heart development is of great interest. Adriana C. Gittenberger-de Groot and colleagues contributed extensively to this end, both characterizing the different cellular sources of the developing heart [3] and applying this knowledge to clinical goals [4].

**Figure 1:**
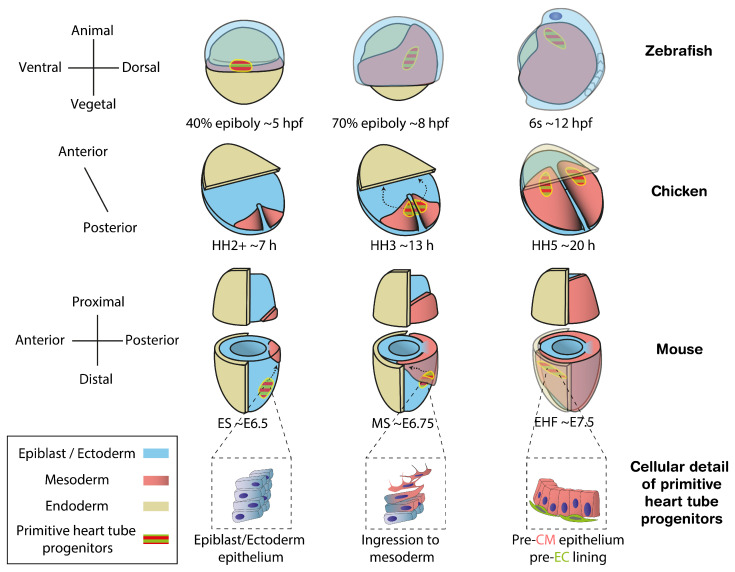
Location of cardiac progenitors in vertebrate models from the onset of gastrulation to the stage preceding primitive heart tube formation. Cardiac progenitors ingress the mesoderm soon after the start of gastrulation, migrating to the opposite side of the embryo to establish the two layers that form the primitive heart tube. The different rows of diagrams show this process in mouse, chicken, zebrafish and a zoom-in for the cellular detail of primitive heart tube progenitors, respectively. Zebrafish diagrams are depicted from dorsal views while those in chick and mouse show ventral views. Morphological staging follows the epiboly rate, Hamburger–Hamilton (HH) and Downs [14] criteria for zebrafish, chicken and mouse, respectively. Approximate time in hours post fertilization (hpf), hours (h) or embryonic day (E) are also provided. CM, cardiomyocytes; EC, endocardium. Dashed arrows depict the migration trajectory of primitive heart tube progenitors during gastrulation.

A key step towards understanding organogenesis is finding how stem cells commit to different cell types. Studying the embryonic origin of the distinct cell populations contributing to an organ helps us to understand developmental defects by identifying within heterogeneous progenitor populations those contributing to specific cellular compartments and functions. In vertebrates, cardiac progenitors in the epiblast are among the earliest to ingress through the primitive streak and differentiate into mesoderm [5,6,7,8] (Figure 1). At this point, the transcription factor *Mesp1* is transiently expressed, offering an accurate marker for nascent mesodermal cells including extrambryonic, cardiac and head/neck skeletal muscle progenitors [9]. Once they have migrated to the anterior pole, a subset of cardiac progenitors, known as the First Heart Field (FHF), starts expressing specific differentiation markers. By Early Head Fold (EHF) stage in mouse, embryonic day (E) ∼7.5, the pre-myocardium is arranged as a columnar epithelium and the pre-endocardium lays between the pre-myocardium epithelium and the endoderm, revealing the first sign of cellular heterogeneity in the heart [10] (Figure 1, cellular detail of primitive heart tube progenitors) [11]. Subsequently, the pre-myocardial epithelium separates from the endoderm, creating bilateral hemi-tube structures with endocardial cells in its lumen. This primordium, known as the cardiac crescent in the mouse, undergoes morphogenesis fusing at the midline to form the primitive heart tube, which will contribute to the left ventricle and part of the atria in the mature heart. Continuous with the primitive heart tube, additional cardiac progenitors, known as the Second Heart Field (SHF), remain undifferentiated until recruited later to the right ventricle, outflow tract and the rest of the atria [12,13].

In this review, we dissect the earliest developmental events that contribute to generate the cellular heterogeneity and complexity of the heart. As other excellent publications review these aspects at later stages in both mammal [1,15,16] and zebrafish models [17], here we will focus on the formation of the primitive heart tube, using the mouse model as a reference. From classic labelling experiments (Table 1) to recent single-cell transcriptomic analysis [18,19], we review studies that have expanded our views on cardiac heterogeneity and the mechanisms that generate it. Finally, we discuss the embryological relevance of such complexity, providing an overview of the public datasets that are available to study it further (Table 2).

Link list (accessed on 25 December 2021):

1. Raw data https://www.ebi.ac.uk/arrayexpress/experiments/E-MTAB-4026/; Browser http://gastrulation.stemcells.cam.ac.uk/scialdone2016
2. Raw data https://www.ncbi.nlm.nih.gov/geo/query/acc.cgi?acc=GSE100471; Browser http://singlecell.stemcells.cam.ac.uk/mesp1
3. Raw and processed data https://www.ebi.ac.uk/arrayexpress/experiments/E-MTAB-6153/
4. Raw data https://www.ebi.ac.uk/arrayexpress/experiments/E-MTAB-6967/; Processed data https://github.com/MarioniLab/EmbryoTimecourse2018; Browser https://marionilab.cruk.cam.ac.uk/MouseGastrulation2018/
5. Raw data https://www.ncbi.nlm.nih.gov/geo/query/acc.cgi?acc=GSE126128; Browser https://cells.ucsc.edu/?ds=mouse-cardiac
6. Raw data https://www.ncbi.nlm.nih.gov/geo/query/acc.cgi?acc=GSE109071
7. Raw data https://www.ncbi.nlm.nih.gov/geo/query/acc.cgi?acc=GSE108963
8. Raw and processed data https://www.ebi.ac.uk/ena/browser/view/PRJEB23303?show=reads
9. Raw and processed data https://www.ncbi.nlm.nih.gov/geo/query/acc.cgi?acc=GSE169210; Browser https://tanaylab.weizmann.ac.il/embflow/
10. Raw, processed data and Browser https://marionilab.cruk.cam.ac.uk/heartAtlas/;
11. Raw data https://www.ncbi.nlm.nih.gov/geo/query/acc.cgi?acc=GSE176306; Browser https://cells.ucsc.edu/?ds=chi-10x-mouse-cardiomyocytes
12. Raw and processed data https://www.ebi.ac.uk/ena/browser/view/PRJEB23303?show=reads;
13. Raw and processed data https://www.ncbi.nlm.nih.gov/geo/query/acc.cgi?acc=GSE133244; Browser https://gottgens-lab.stemcells.cam.ac.uk/snATACseq_E825/
14. Raw data https://www.ncbi.nlm.nih.gov/geo/query/acc.cgi?acc=GSE121708; Processed data ftp://ftp.ebi.ac.uk/pub/databases/scnmtgastrulation
15. Raw data https://idr.openmicroscopy.org/webclient/?show=project-502

## 2. Cell Fate Specification Preceding Primitive Heart Tube Formation

Once the primitive heart tube is assembled, it contains two cell types: cardiomyocytes (CMs), which form the muscular wall and endocardial cells (ECs), which are specialized endothelial cells lining the cardiac lumen [41]. Besides forming a continuum with the embryonic vasculature, ECs are involved in the formation of trabeculae [42,43,44] and contribute to the formation of the cardiac valves and septa [45,46]. ECs differ from other endothelial cells in their gene expression profile [47,48,49] and follow specific differentiation programs [42]. In the course of evolution, CMs arose from the transformation of mesenteric coelomic epithelium in early metazoans [50] while endothelial cells appeared later in vertebrates, likely from adherent hemocytes [51]. With these phylogenetic differences, CMs and ECs are found side-by-side at the anterior splanchnic mesoderm at the initiation of heart tube formation (Figure 1). Their ontogeny has been studied across multiple organisms, but it remains unclear if they originate from a homogeneous pool or from two distinct populations, pre-specified to adopt CM or EC fate [11,52].

### 2.1. Temporal Sequence of Fate Acquisition

Knowing when a fate decision takes place is the first step to understand the mechanisms governing it. In classic embryology, a single cell or a group of cells is considered *specified* when it systematically yields a certain cell type. A tool to address the temporal sequence specification is clonal analysis [53]. By labelling single cells at different developmental stages and examining their progenies, one can infer when cell lineages become restricted—i.e., a progenitor cell that gave rise to both CMs and ECs was not specified to either fate at the time it was labelled. In prospective clonal analysis, researchers know the stage and location of the progenitor cell. This is achieved by direct labelling through manipulation of oviparous or *ex utero* viviparous embryos. In retrospective clonal analysis, cells are genetically labelled *in utero* so the embryonic stage and cell location are only approximated or unknown [54].

Clonal analysis experiments in zebrafish, chicken and mouse models traced the location of cardiovascular progenitors and pointed to an early specification of CMs and ECs, happening around the onset of gastrulation. To ease the interpretation of these valuable data, we summarized the experiments in Table 1. Prospective labelling in zebrafish early blastula (512 cells, 2.75 hpf) defined an area at the lateral marginal zone, enriched for unspecified cardiovascular progenitors [20]. Labelling the same area at 40% epiboly stage (5 hpf) yields few mixed progenies containing both CMs and ECs, indicating they are already specified when gastrulation begins [21]. Fate mapping in both cases shows that CM and EC progenitors are spatially intermingled. In chicken embryos, the rostral half of the primitive streak contains both cardiac progenitors [55,56], which are already specified to form either CM or EC progenies [23,57]. Retrospective clonal analysis in mouse embryos also suggests an early specification. Genetically labelled *Mesp1*-expressing cells around the onset of gastrulation (∼E6.25) give rise to clusters in the left ventricle at E14.5 containing either only CM or only EC [24]. Conversely, a quarter of *Mesp1*-expressing clones labelled at ∼E7.25 gave rise to mixed progenies in the right ventricle [24], indicating that although the primitive heart tube arises from precursors that are already specified at gastrulation, later pools of progenitors from the second heart field can be multipotent [58]. In line with this result, lineage tracing and in vitro studies have suggested the existence of mouse [58,59,60,61,62] and human [63] multipotent cardiac progenitors. A way to characterize further these progenitors would be to examine recent scRNAseq datasets for a cardiac multipotency signature in gastrulating mouse [18,28,29] and human embryos [64]. Together, these studies show CM and EC specification occurs at the onset of gastrulation or even before across vertebrate species, which is surprisingly long before the start of heart morphogenesis; however, pools of cardiac progenitors established later at the second heart field may remain multipotent.

Regardless of the timing of specification, the lineage relationship between CMs and ECs is controversial. Genetic lineage tracing of cardiac transcription factors and stem cell experiments support both cell types arise from a cardiac-specific common progenitor [11,59,60,61,65,66,67]. In contrast, zebrafish mid-blastula clones containing both CMs and ECs also give rise to blood vessels and blood cells [20] (Table 1), suggesting a CM-EC exclusive progenitor does not exist or it does very transiently. An alternative lineage tree proposes that ECs derive from the hematopoietic/vascular progenitors, and then migrate to populate the developing heart tube in zebrafish embryos [68,69]. To assess the lineage relationship among cardiac, hematoendothelial and other mesodermal progenitors, future clonal analysis experiments should examine the presence of labelled cells in all mesodermal tissues and not only in the heart [19]. New approaches such as CRISPR/Cas9-based lineage tracing [70,71] and *in toto* live-cell tracking [38], will also help to answer this long-standing question.

### 2.2. Molecular Mechanisms of Specification

Understanding the mechanisms underlying cell fate decisions involves characterizing the cellular heterogeneity that precedes lineage specification [72]. A homogeneous niche of progenitors can segregate by various mechanisms to form subpopulations with different fates. Recently, the development of single cell transcriptomics and genomics transformed the way we study cellular heterogeneity in vivo [28,37,73]. The simultaneous characterization of the different cell populations forming an embryo allows one to make predictions about their ontogeny as well as showcasing gene regulatory networks responsible for it [74].

Single-cell RNA sequencing (scRNAseq) of gastrulating *Mesp1*-positive cells displays the transcriptional divergence of cardiac progenitors in vivo, revealing that CM and EC branches first diverge at ∼E7.25 [26]. If pre-specified CM and EC progenitors exist at PS ingression (∼E6.75 in mouse)—see Section 2.1—the transcriptional divergence captured at ∼E7.25 by this approach may account for the earliest signs of differentiation but it remains unknown whether this segregation is a direct effect of their prior specification. Analyzing the trajectory of cardiac cells in emerging scRNAseq datasets (Table 2), especially in those prioritizing sequence depth over cell number, would shed light on whether transcriptional differences account for CM and EC early specification.

As demonstrated in *Drosophila*, single-cell chromatin accessibility mapping also allows identifying molecular processes involved in fate specification [75]. In addition to differences in RNA expression, epigenetic modifications contribute to cellular heterogeneity. As an example, early haematoendothelial clusters defined by scRNAseq in mouse can be further classified by their open chromatin regions corresponding to *Tal1*-bound cell-type specific enhancers [76]. Similarly, chromatin accessibility mapping in mouse ∼E8.5 *Isl1*-expressing cells reveals distinct epigenetic signatures, likely corresponding to differently fated progenitors [32]. This shows how cells within a scRNAseq cluster can differ in terms of accessibility to regulatory loci that account for changes in responsiveness to signaling cues. Thus, epigenetic analyses may distinguish fate specification in transcriptionally homogeneous populations, as these changes can anticipate RNA expression divergence. In fact, analysis of poised enhancers—distinguished from active enhancers by the H3K27me3 mark [77]—predicts developmental competence in human derived endodermal stem cells [78]. This, together with the functional relevance of chromatin remodeling complexes in gastrulation and cardiogenesis [79,80] makes epigenetic heterogeneity a candidate mechanism to explain CM and EC early segregation in primitive heart tube formation. The increasing availability of single-cell transposase-accessible chromatin (scATACseq) methods [34] will likely motivate researchers to explore epigenetic heterogeneity in the coming years.

## 3. Differentiation of Primitive Heart Tube Progenitors

### 3.1. Signaling Cues Driving CM and EC Differentiation

After ingression, CM and EC progenitors migrate to the anterior-proximal side of the embryo proper (see Section 1 and Figure 1), where signaling cues from the subjacent endoderm promote their differentiation [81,82]. Integration of the BMP, FGF and Wnt pathways forges an environment that promotes primitive heart tube morphogenesis among vertebrate species [83]. For example, *Bmp2* and *Fgf8* zebrafish mutant embryos express less *Nkx2-5* and differentiate fewer CMs. Removal of endoderm also causes a downregulation of cardiac markers in chicken, which can be rescued by supplying exogenous FGF8 or BMP2 [84]. In vitro, BMP2 released from anterior visceral endoderm cell lines induces CM generation in embryoid bodies [85]. On the other hand, Wnt/β-catenin signaling prevents premature CM differentiation at the lateral plate mesoderm, but its expression is necessary for CM progenitor proliferation and ingression through the primitive streak [86]. In vitro, the timing of Wnt activation/deactivation cycles is also critical for CM differentiation in human induced pluripotent stem cells [87] and heart organoids [88]. Likewise, *Wnt5a*-mediated Wnt inhibition promotes EC differentiation in mouse early cardiac progenitors, while hindering CM differentiation [89]. Altogether, the signaling environment provided by the endoderm ensures that CM and EC differentiation occurs at the right time and location. In fact, the anterior intestinal portal can induce cardiac identity from non-cardiac mesoderm and pattern the ventricular and atrial domains in chicken, pinpointing the anterior endoderm as a heart organizer in vertebrates [90].

Besides diffusible cues, local signaling also plays a role in CM and EC differentiation. For example, cells with active Notch do not form CM colonies and forced activation of NOTCH1 in embryonic stem (ES) cells inhibits their differentiation to CM, while Notch inactivation promotes it [91,92]. This inhibitory effect also takes place in vivo: in Drosophila, loss and gain of function studies show Notch inhibits CM differentiation [93]; in Xenopus, Notch signaling limits the number of CM through the Serrate ligand [94]; in chicken, retroviral overexpression of Notch intracellular domain (NICD) in the heart tube reduces the expression of CM markers [95]; and in mouse, although NICD overexpression does not alter the number of CM or marker expression, it results in CM maturation defects including disrupted sarcomeric structures [96]. Conversely, *Notch1* is required for the development of the endothelium [97,98] and its expression marks mesodermal progenitors differentiating towards EC [26].

Unlike that of endothelial cells, CM differentiation is tightly coupled to gastrulation. Removal of the transcription factors *Etv2* or *Npas4l* yields embryos that lack endothelial cells but undergo gastrulation normally, forming a heart tube without ECs [99,100]. To date, no genetic manipulation produced embryos that gastrulate but fail to form CMs: all mutants without CMs also fail to gastrulate, lacking all anterior mesoderm tissues [101,102,103,104,105,106]. In some of these, stuck mesodermal cells express CM differentiation markers [106] or even form bilateral heart tubes [9]. This suggests that once gastrulation is initiated successfully, CM differentiation will occur regardless of the anterior endoderm signaling cues [107]. In fact, gastrulation and early heart tube formation share many common genetic cascades implicating members of the *Mesp* transcription factor, Fgf and Wnt signaling pathways. An interpretation is that differentiation towards CM is determined concomitantly with gastrulation, or even represents the default state of anterior mesoderm, and the signaling environment may only modulate when and where the differentiation takes place. In support of this notion, non-cardiac mesoderm regions need to repress the CM programs to avoid ectopic differentiation [69,108,109,110].

### 3.2. Redirection of Cardiac Progenitor Differentiation upon Perturbation

Although primitive heart tube progenitors get specified to CMs and ECs at early stages (see Section 2.1), their definitive differentiation depends on surrounding signaling cues (see Section 3.1) and can be modified upon perturbation. In zebrafish, embryos lacking transcription factors as *Tal1* and *Etv2* have fewer ECs but expand their CM pool [42,108], with some *Etv2* endothelial progenitors differentiating to CMs [108]. Indeed, scRNAseq shows FGF and Wnt signaling detours vascular progenitors towards a muscular fate in the absence of *Etv2* [111]. In line with this, injecting *Tal1* or *Etv2* mRNA at the lateral plate mesoderm expands the endothelial domain while reducing the number of CMs [69]. In mouse, deletion of *Tal1* yields CM-like differentiation in both yolk sac and endocardium through cell-autonomous Wnt antagonism [112]. Likewise, the enforced activation or inhibition of Wnt reduced or increased, respectively, endothelial differentiation in cardiac progenitors [89]. Finally, overexpression of *Sox17*, which is expressed downstream of *Etv2*, causes ectopic expression of PECAM1 endothelial marker in CMs [113].

Overall, these studies show CM and EC progenitors can modulate their fate in vivo upon perturbation of differentiation pathways, redirecting their fate even after specified. In vitro differentiation studies also illustrate the versatility of early mesodermal progenitors. A subset of ES-derived mesodermal cells, which start expressing *Flk1*, can differentiate to either CMs or ECs in a context-dependent manner [59,61]. Notably, a sub-population of the *Flk1*-negative ES-derived mesodermal cells can also be redirected in vitro towards a myocardial cell fate in mouse and human [114,115,116]. However, these results must be interpreted with caution, as stem cells can take differentiation roads that are not developmentally relevant.

### 3.3. Plasticity of Cardiac Progenitors

In early cardiac progenitors, cellular plasticity has been reported in vivo after primitive heart tube formation [1,117] and in ES cells models [118]. An example of in vivo cell plasticity is the interchangeability between atrial and ventricular CM progenitors. In chicken, cardiac ventricle progenitors derive from anterior regions of the cardiac mesoderm while atria progenitors arise from more posterior regions [55,119,120,121,122]. However, presumptive atrial cells can adopt ventricular properties when placed in the prospective ventricular domain [123] up to HH8 stage—when heart tube assembles. In mouse embryos, deletion of *COUP-TFII* in atrial CMs can switch their identity to ventricular CMs up to stage ∼12.5, long after the formation of the cardiac chambers [124].

Together, the experiments cited in these three subsections show the definitive differentiation of cardiac progenitors is not fixed as it can change upon positional cues or intrinsic signaling perturbations. This way, despite the mechanisms normally involved in establishing the identity of cardiac cells, the signaling environment plays a role in their definitive differentiation [125].

## 4. Molecular Heterogeneity of the Cardiomyocyte Sources within the Primitive Heart Tube

**Figure 2:**
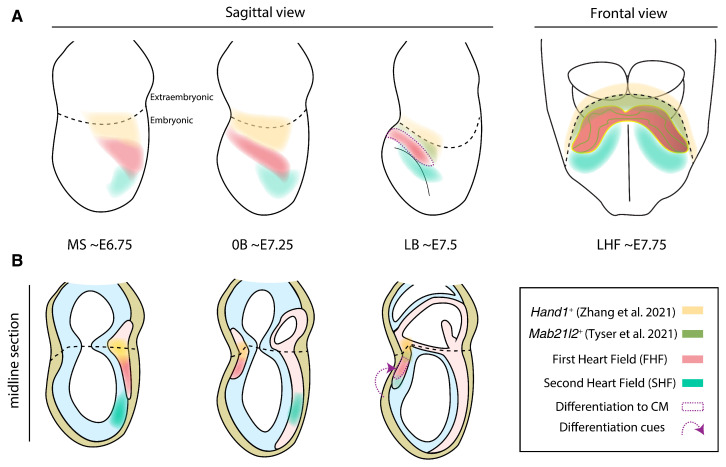
Progenitor domains contributing to heart tube cell populations in the mouse. Diagrams show whole embryos (**A**) and midline sections (**B**). Notice how the First Heart Field (FHF), Second Heart Field (SHF) and the recently characterized *Mab21l2*/*Hand1* population stay in a continuum until differentiation cues recruit the FHF to form the primitive heart tube. In the section diagrams (**B**), endoderm, epiblast and mesoderm are painted in yellow, cyan and light red, following Figure 1 color code. Diagrams were done based on [18,19].

Studying the molecular regulation of the different cell populations composing the primitive heart tube is important to understand their subsequent role in morphogenesis. Molecular signatures give us hints about cell behavior, features and susceptibility to signaling cues. Two recent outstanding papers have characterized the primitive heart tube transcriptional heterogeneity with unprecedented detail, reporting a novel cardiac progenitor pool that contributes to FHF cardiomyocytes and contains the earliest known progenitors of the epicardium [18,19] (Figure 2).

Single-cell RNA sequencing (scRNAseq) of the mouse anterior cardiac region at cardiac crescent to linear heart tube stages (∼E7.75 to ∼E8.25) identified six different cardiac clusters in the anterior-proximal region of the embryo proper [18]. Differential expression analysis linked two of the clusters to the first and second heart field, respectively, while a third cluster represented an intermediate differentiation state between both. Strikingly, one of the clusters did not fit any previously known categorization, as it expressed some FHF markers, like *Hand1* and *Tbx5*, but lacked canonical differentiation markers, such as *Nkx2-5*. RNA fluoresence in situ hybridization of marker *Mab21l2*, a protein coding gene implicated in cardiac and neural development [126,127], mapped this novel cluster at the rostral border of the cardiac crescent, forming a narrow band of splanchnic mesoderm at the confluence of the embryonic and extraembryonic compartments of the embryo (Figure 2).

Analysis of the scRNAseq data revealed two distinct trajectories towards differentiated CMs, parting from the SHF cluster and the *Mab21l2* cluster, respectively; the first connected SHF to differentiated CM via an intermediate state, which likely corresponds to the incorporation of SHF progenitors to the heart tube at the arterial pole [13,128,129]; the second trajectory linked the newly identified *Mab21l2* cluster to differentiated CM via another intermediate state, unveiling a previously undescribed source of CMs. Lineage tracing of cells expressing *Mab21l2* at ∼E6.5–E7.5 yielded left ventricle CMs as well as epicardial cells. However, this cluster does not contribute to CMs of the right ventricle or outflow track. This implies that the *Mab21l2* cluster, dubbed Juxta Cardiac Field (JCF) by the authors, supplies CMs to the FHF and contains the earliest known progenitors of the epicardium.

With a similar strategy, scRNAseq and trajectory analysis of *Mesp1*-expressing cells from ∼E7.25–E8.25 mouse embryos also predicted a cardiac population [19] that partially overlaps the *Mab21l2* cluster [18] but expands beyond the extraembryonic boundary (Figure 2). Lineage tracing of *Hand1*-expressing cells at ∼E5.75–E6.75 yielded a contribution to the heart tube similar to that of the *Mab21l2* cluster [18], reinforcing the idea of the cardiac potential this cell population. In addition, retrospective clonal analysis of *Hand1*-expressing cells at ∼E6.75–E8.25 revealed that these progenitors are multipotent. While most progenies contributed only to the yolk sac, a fourth contained a mixture of two or three distinct lineages including yolk sack, pericardium, proepicardium and atrioventricular canal or left ventricle CM [19]. As retrospective analyses render only approximate staging, multipotent clones may have resulted either from early inductions at the primitive streak or from later mesodermal cells that cross the embryonic/extraembryonic boundary. The latter would disagree with live imaging data reporting a clear segregation between embryonic and extraembryonic cells following the onset of gastrulation [39]. Estimating the induction time of the clones from their cell counts or prospectively labelling the *Hand1* domain would clarify for how long this novel population remains multipotent.

Altogether, these studies uncover a previously uncharacterized cardiac progenitor population contributing mainly to the left ventricle and epicardium. In both publications, this population lies at the embryonic/extraembryonic boundary and constitutes a spatially and transcriptionally distinct population from *Tbx18*-expressing sinus venosus progenitors, which are recruited from a more caudal splanchnic mesoderm area [130]. While the first study defined this population at cardiac crescent stages (∼E7.75) using *Mab2l12* as a marker [18], the second described a broader and earlier population expressing *Hand1* at ∼E6.25–E7.25 [19], making the *Mab21l2* domain a likely subset among *Hand1*-expressing cells (Figure 2). Whether this population remains multipotent at cardiac crescent stages or only holds this ability at earlier stages is a pending question.

## 5. Discussion and Future Perspectives

Early heart development is a complex process involving heterogeneous sources of cell progenitors. Such complexity allows the heart to function while it keeps forming but makes the process susceptible to errors, likely responsible for the high incidence of congenital heart defects. Although single-gene mutations are linked to certain rare diseases [131], understanding the wide spectrum of congenital heart defects requires the integration of multiple gene regulatory networks that pattern the heterogeneous set of cellular functions in the heart.

An early lineage diversification in cardiac development emerges around gastrulation, when progenitor cells specify towards myocardial and endocardial fates. Chicken and zebrafish prospective clonal analysis demonstrate CM and EC progenitors are already segregated once they are recruited to the mesoderm, with retrospective experiments in mouse suggesting a similar outcome (see Section 2.1). However, it is still unclear when this fate decision takes place and what are the mechanisms governing it. Classic precepts consider that a common cardiac progenitor bifurcates into either CM or EC, but alternative models suggest ECs derive from a common endothelial lineage, shared with the rest of the vessels in the embryo proper and yolk sac (see Section 2.1). As the endothelium is a relatively recent innovation in evolution, this raises the question of whether early vertebrates recycled the CM program in the mesoderm to form endothelial cells and build complex circulatory systems. A dedicated analysis of single-cell genomics and live imaging data (Table 2) will likely define the ontogeny of CM and EC populations, charting the definitive Waddington landscape [132] of primitive heart tube specification. Interpretation of the big data generated by these approaches requires the use of programming and mathematics [133], but step-by-step tutorials allow researchers from a life sciences background to access these tools [40]. In fact, an increasing number of papers are setting an example by providing user-friendly guidance on the use of their code, easing the reanalysis of their data [28,38].

In a broader view, these high-resolution data are reshaping our perspectives on how embryos develop. In classic embryology textbooks, embryos were classified into two broad categories: regulative and mosaic embryos [134]. In the first type, characteristic of vertebrates, cells organize to form different organs by regulating their fate “on the go” through interactions with their surroundings. Regulative embryos can adapt to perturbations as cell decisions are continuously rechecked according to positional information and the signaling environment. In mosaic embryos, characteristic of the invertebrates, each cell or group of cells have a restricted fate, which is acquired autonomously through intrinsic factors. A century later, we know that in reality all embryos are both mosaic and regulative to some extent. Ascidian and nematode embryos, examples of stereotyped development with invariant cell lineages, also employ cell-cell contacts and cytokine signaling to orchestrate organ differentiation once they reach a certain cell number [135,136]. Conversely, mouse ES cells cannot contribute to some extraembryonic tissues such as the trophoblast and primitive endoderm, suggesting an intrinsic restriction in cell potency in early vertebrate embryos [137]. Thus, as shown in this review for the primitive heart tube, both intrinsic cell heterogeneity and signaling cues collaborate to progressively define cellular identity during embryo development.

In the discussion between regulative and predetermined conceptions, the division of cardiac progenitors as multiple predefined populations is a topic of debate. While some studies show an early segregation of the first and second heart field as predetermined populations with distinct susceptibility to differentiation [24,26,58], others argue that the earlier differentiation of the FHF is governed by positional cues and not by intrinsic cellular differences [138]. Likewise, the recently characterized *Mab21l2*/*Hand1* progenitors contributing to cardiomyocytes and epicardium (see Section 4 and Figure 2) could be defined as a separate cardiac field. In favor of this notion, these progenitors show a unique transcriptomic signature and contribute systematically to specific regions of the heart. Nonetheless, it still remains to be tested whether the contributions of these regions are essential for heart development. In the case of FHF and SHF, elimination of the contribution of either population results in fatal cardiac malformations [139], whereas elimination of the contribution of these newly described regions to the cardiomyocyte pool has not been explored. On the other hand, the definition of developmental fields based solely on the recombination pattern of a particular transgene poses some questions, like whether the transgene labels cells before the full specification of the fields. In any case, the description of these novel *Mab21l2*/*Hand1* progenitors deepens our understanding on the heterogeneous sources of the cells that form the heart tube. A key question that arises is whether these heterogeneous sources confer developmental robustness and functional diversity to the mammalian heart. In that sense, it will be interesting to assess whether the *Mab21l2*/*Hand1* progenitors are also present in anamniotes [17] or instead constitutes an evolutionary novelty that contributed to increase the complexity of the heart tube. Thus, exploiting single-cell omics and live-imaging data will lead to novel insights in understanding heart development (Table 2). The same way pioneer work by Gittenberger-de Groot and colleagues illuminated the different sources contributing to the arterial pole [140], identifying the states of the interacting components of the heart will continue to shed light on our understanding of congenital heart defects.

## Figures and Tables

**Table 1:** Clonal analysis studies assessing early cardiac lineage segregation in vertebrate models.

Model	Methodology	Stage	Location	N	Progeny (% from Total)				Reference
					CM	EC	CM + EC	CM + EC + Non Cardiac	
Zebrafish	Single-cell dextran microinjection	Early blastula (∼2.75 hpf)	lateral-marginal zone	29	4	10	21	65	[20]
Zebrafish	Single-cell dextran microinjection	Midblastula (∼3 hpf )	lateral-marginal zone	41	18	7	0	75	[20]
Zebrafish	2–3 cells fluorescein activation	40% epiboly (∼5 hpf)	ventral-marginal zone	69	44	52	4	ND	[21]
Zebrafish	Kaede photoconversion	14-somite (∼18 hpf)	anterior lateral plate mesoderm	39	27	0	2	ND	[22]
Chicken	Replication-defective retrovirus	HH3 (∼14 h)	anterior lateral plate mesoderm	82	95	5	0	ND	[23]
Chicken	Replication-defective retrovirus	HH4 (∼18 h)	rostral portion primitive streak	36	55 *	45 *	0	ND	[23]
Mouse	Doxycyclin induced transgene expression	Early primitive streak (∼E6.5) **	*Mesp1*-expressing cells	13	85	15	0	ND	[24]
Mouse	Doxycyclin induced transgene expression	Late primitive streak (∼E7.0) **	*Mesp1*-expressing cells	6	100	0	0	ND	[24]
Mouse	Doxycyclin induced transgene expression	Late bud (∼E7.5) **	*Mesp1*-expressing cells	17	70	6	24 ***	ND	[24]

ND, Not Determined; hpf, hours post fertilization. * Including labeled clusters that consisted of both CM or EC but either tagged with cytoplasmic *β*-gal (*β*-gal) or nuclear directed *β*-gal (n*β*-gal). ** Estimated recombination stage. Mouse retrospective analyses can only offer an approximated stage as the precise mating time and litter variability are unknown. *** A third of the clusters also contain smooth muscle cells.

**Table 2:** Single-cell sequencing and live imaging data from mouse embryos available in the literature. Links to the raw and processed data and their website interfaces are provided when available (Data).

Resource	Stage	Selection (N Cells)	Method	Depth *	Data	Reference
scRNAseq	ES to LHF ∼E6.5, E7.5, E7.75	∼E6.5 epiblast (501) ∼E7.5 Flk1^+^ (704)	Smart-seq2	∼1 × 10^6^ reads/cell	link list 1	[25]
scRNAseq	∼E6.75, E7.25	Mesp1^+^ ∼E6.75 (83 WT 85 Mesp1^−/−^) ∼E7.25 (173)	Smart-seq2	∼1 × 10^6^ reads/cell **	link list 2	[26]
scRNAseq	∼E8.25	Whole embryo (19,396)	10× genomics	∼2 × 10^4^ UMIs/cell	link list 3	[27]
scRNAseq	ES to 7 s ∼E6.5, E6.75, E7.25, E7.5, E7.75, E8.0, E8.25, E8.5	Whole embryo (116,312) Per stage: supplementary	10× genomics	∼2 × 10^4^ UMIs/cell	link list 4	[28]
scRNAseq	4 s, 8 s, 21 s ∼E7.75, E8.25, E9.25	Dissected cardiac region E7.75 (4326 WT 3535 Hand2^−/−^) E8.25 (5664 WT 4112 Hand2^−/−^) E9.25 (11,376 WT)	10× genomics	∼2 × 10^4^ UMIs/cell	link list 5	[29]
scRNAseq	LHF, 8 s, 13 s, 20 s ∼E7.75, E8.25, E8.75, E9.25	Nkx2-5^+^ (690), Isl1^+^ (640) Per stage: supplementary 1	Modified Smart-seq2	∼1 × 10^6^ reads/cell	link list 6	[30]
scRNAseq	Pre-Streak stages ∼E5.25, E5.5, E6.25, E6.5	Whole embryo E5.25 (331), E5.5 (269) E6.25 (321), E6.5 (803)	Smart-seq2	∼1 × 10^6^ reads/cell **	link list 7	[31]
scRNAseq	∼E7.5, E8.5, E9.5	Nkx2-5^+^ E7.5 (61), E8.5 (58) E9.5 (81) Isl1^+^ E7.5 (30), E8.5 (167) E9.5 (348 WT 50 Isl1^−/−^)	Smart-seq2	∼1 × 10^6^ reads/cell **	link list 8	[32]
scRNAseq	PrS to Presomitic ∼E6.5 to E8.25	Whole embryo (33,700 from 153 embryos)	MARS-seq	∼4 × 10^3^ UMIs/cell	link list 9	[33]
scRNAseq	LHF to 4 s ∼E7.75 to E8.25	dissected cardiac region (3105)	Smart-seq2	∼1 × 10^6^ reads/cell **	link list 10	[18]
scRNAseq	0B to somite stage ∼E7.25 to E8.25	Mesp1^+^ (9072)	10× genomics	60,450 UMIs/cell	link list 11	[19]
snATACseq	∼E8.5, E9.5	Isl1^+^ (695)	[34]	∼1.5 × 10^4^ reads/nucleus *	link list 12	[32]
snATACseq	∼E8.25	Whole embryo (19,453)	[35]	∼2 × 10^4^ reads/nucleus *	link list 13	[28]
scNMTseq	∼4.5, E5.5, E6.5, E7.5	Whole embryo (856)	[36]	∼1 × 10^6^ reads/cell ***	link list 14	[37]
Live imaging	LB to 4 s ∼E7.5 to E8.5	Cardiac region: 4 embryos Tdtomato mosaic Nkx2-5:GFP	Two-photon microscopy	10 min 5 μm	NA	[13]
Live imaging	LS to 2 s ∼E7.0 to E8.25	4 Whole embryos, H2B:eGFP	Adaptative light-sheet microscopy	4 min 2 μm	link list 15	[38]
Live imaging	MS to LB ∼E6.75 to 7.5	4 Whole embryo: T-cre mT/mG mosaic	Two-photon microscopy	20 min 3 μm	NA	[39]

NA, not available; s, somite pairs. * Median reads or time-frame and z size are shown for scOmics or live imaging experiments, respectively. In scRNAseq, lower depth approaches amplify cDNA before sequencing to increase sensitivity. Before that, captured molecules are labeled with a unique molecular identifier (UMI). For more info read [40]. ** Value not provided, calculated from the counts matrix. *** Corresponding to scRNAseq.

## Data Availability

Not applicable.
